# Comparative study on the rumen microbial communities and functions between Wagyu and Holstein calves

**DOI:** 10.1186/s12864-025-12392-1

**Published:** 2025-12-04

**Authors:** Ye Bu, Fang Sun, Li Liu, Xinmiao He, Haoran Wang, Zhaohui Chen, Tengfei He, Shanshan Xu, Xiaochuan Zhao, Xiangren Meng

**Affiliations:** 1 Institute of Animal Husbandry of Heilongjiang Academy of Agricultural Sciences, Harbin, 150086 China; 2 College of Animal Science and Technology, Northeast Agricultural University, Harbin, 150030 China; 3 College of Animal Science and Technology, China Agricultural University, Beijing, 100083 China

**Keywords:** Wagyu, Holstein, Calves, Rumen microbiota, Metagenomics

## Abstract

**Background:**

Understanding the rumen microbiota’s development in calves is essential for optimizing breed-specific feeding strategies. This study aimed to comparatively investigate the dynamic changes in the rumen microbial community structure and function in Wagyu and Holstein calves.

**Methods:**

Five 3-month-old Wagyu calves and five age-matched Holstein calves were selected. All animals received the same diet consisting of concentrate and hay, with free access to feed and water. Rumen fluid samples were collected monthly from 3 to 6 months of age. Metagenomic sequencing was performed to assess microbial composition (phylum and genus levels), alpha diversity (Shannon, Simpson, ACE, and Chao1 indices), and functional pathway (KEGG-based).

**Results:**

The cumulative relative abundance of dominant taxa at both phylum and genus levels declined with age in both breeds, more markedly in Wagyu calves than in Holsteins. From 3 to 6 months of age, the top five phyla combined dropped by 3.25% in Wagyu and 0.87% in Holstein calves, whereas the top ten genera combined decreased by 1.63% and 0.63%, respectively. Alpha diversity in Wagyu calves increased significantly with age. At 5 and 6 months, the Shannon, ACE, and Chao1 indices were significantly higher than those at 3 months (*P* < 0.05). Moreover, from 4 to 6 months, Wagyu calves consistently exhibited significantly higher diversity indices than Holsteins (*P* < 0.05). At 6 months, Wagyu calves showed a significant reduction in metabolism-related microbial genes and an increase in genes related to cellular processes and genetic information processing compared to earlier ages and Holstein calves (*P* < 0.05).

**Conclusions:**

These findings suggest potential breed-specific differences in the succession and functional maturation of rumen microbiota. Holstein calves developed earlier and more stable metabolic functions, while Wagyu calves underwent a more dynamic microbial selection process.

**Clinical trial number:**

Not applicable.

**Supplementary Information:**

The online version contains supplementary material available at 10.1186/s12864-025-12392-1.

## Background

The rumen microbiota plays a pivotal role in the digestion and nutrient metabolism of ruminants [1]. As a complex and dynamic microbial ecosystem, it facilitates the breakdown of plant fibers and the synthesis of volatile fatty acids (VFAs) and microbial protein, which are critical for the host’s energy supply and growth [2, 3]. The structure and function of the rumen microbial community are known to be influenced by multiple factors, including diet, age, and host genetics [4]. Notably, breed-specific characteristics may shape distinct trajectories in microbial succession and metabolic capacity. However, while such differences have been explored in adult cattle, there is limited knowledge regarding how rumen microbiota evolve in calves, especially during the critical developmental window from weaning to early rearing.

Current research largely focuses on the rumen microbiota of mature ruminants [5], often overlooking the calf stage as merely preparatory. Yet, the establishment and functional maturation of the rumen microbiome during early life are crucial for the long-term productivity of the animal [6, 7]. This period represents a window of microbial imprinting that can influence feed efficiency, growth rate, and even lipid metabolism later in life [8]. Given the divergent breeding objectives, i.e. meat production in Wagyu cattle [9] and milk production in Holstein cattle [10], it is plausible that their rumen microbial communities develop along different trajectories. However, comparative longitudinal studies investigating these dynamics in calves remain scarce, limiting the capacity to design breed-specific nutritional strategies.

To address this gap, the present study aimed to systematically compare the development of rumen microbial communities and functions in Wagyu and Holstein calves from 3 to 6 months of age. Using a metagenomic approach, we tracked changes in microbial community composition, diversity indices (e.g., Shannon, Simpson, ACE, and Chao1), and functional genes abundance across key pathways related to metabolism and genetic information processing. A 120-day parallel-breed design was employed to capture both temporal changes and interbreed differences during this formative stage.

We hypothesized that due to their genetic backgrounds and differing physiological demands, Wagyu and Holstein calves would exhibit breed-specific patterns in microbial succession and functional maturation. Specifically, we sought to address the following questions: (1) Do dominant microbial taxa and diversity indices follow distinct breed-dependent trends with age? (2) Are temporal changes in putative functional genes and pathways enrichment—such as those involved in carbohydrate metabolism or cellular processes—aligned with the production-oriented traits of each breed? This study offers both theoretical and practical value. Theoretically, it deepens our understanding of host–microbiota co-development in calves, providing insights into the mechanisms driving microbial succession under genetic and nutritional influences. Practically, the results may inform precision nutrition and microbiota-targeted interventions, supporting improved early growth performance and sustainability in calf fattening of breeds such as Holstein and Wagyu.

## Materials and methods

### Experimental design and diet

The experiment was conducted from August to November 2024. Five clinically healthy Wagyu calves (Group W) and five Holstein calves (Group H), all aged 3 months at the start of the trial, were included in the study. The Wagyu calves were reared at Zhaoyuan Hongji Zhihe Livestock Breeding Co., Ltd., and the Holstein calves were reared at Heilongjiang Beidahuang Jiusan Animal Husbandry Development Co., Ltd. The initial weight and age of the animal are shown in Table 1. All animals were raised individually at their respective farms under consistent feeding and management conditions, ensuring comparable environmental exposure and adherence to the technical protocols of each farm. Data on feed intake and nutrient levels of all animals are shown in Table 2. Importantly, the animals were not newly purchased for the study but were part of the farms’ routine herd management.

**Table 1:** The initial age and weight of the animals

Breed	Number	Initial Age (days)	Initial Weight (kg)	Breed	Number	Initial Age (days)	Initial Weight (kg)
Wagyu	1	94	82.50	Holstein	1	97	101.60
	2	96	81.10		2	105	96.90
	3	89	84.60		3	105	95.10
	4	94	86.10		4	108	99.10
	5	106	81.20		5	107	98.90

**Table 2:** Data on feed intake and nutrient levels of all animals

Items	Content					
	3 to 4 months		4 to 5 months		5 to 6 months	
Ingredients	Average daily feed intake (kg/head/day)	Percent	Average daily feed intake (kg/head/day)	Percent	Average daily feed intake (kg/head/day)	Percent
Supplementary concentrate for calves (TD660)^a^	2.00	62.50	2.80	66.67	3.60	67.92
Steam-flaked corn	1.00	31.25	1.10	26.19	1.20	22.64
*Aneurolepidium chinensis* ^b^	0.20	6.25	0.30	7.14	0.50	9.43
Total	3.20	100	4.20	100	5.30	100
Nutrient levels^c^						
Moisture	6.88		7.32		7.66	
Crude Protein	19.24		20.46		20.68	
Ether Extract	7.15		8.37		8.48	
Neutral Detergent Fiber	31.07		32.14		32.08	
Acid Detergent Fiber	19.15		19.37		20.36	
Crude Ash	8.27		9.03		9.33	
Calcium	1.58		1.61		1.63	
Total Phosphorus	0.80		0.85		0.84	

^a^Ingredients for TDD660: Soybean meal, Yeast culture, Expanded soybean, DDGS, Corn germ meal, Soybean Oil, Molasses, Dicalcium phosphate, Vitamin A acetate, Vitamin D_3_, Vitamin E, NaCl, Dietary Lysine, Methionine metabolism, Trace elements, et al. The concentrate formula is proprietary, the manufacturer could provide only indicative nutrient reference values: Crude Protein (23.0%), Lysine (1.0%), Crude Fiber (10.0%), Crude Ash (12.0%), Moisture (13.5%), Sodium Chloride (0.5%), Calcium (1.0%), Total Phosphorus (0.6%)

^b^Values are means after ad libitum feeding of Aneurolepidium chinensis

^c^Nutrient levels were measured values

The total experimental period lasted 105 days, consisting of a 15-day pre-feeding phase that commenced when the calves reached 3 months of age, during which early weaning was completed, followed by a 90-day formal trial period. During the entire trial, all calves received the same commercial calf concentrate supplement (TD660) and *Aneurolepidium chinense* (sheep-grass), with free access to both feed and water.

Sampling and assessments were performed at 3, 4, 5, and 6 months of age for both breeds, designated as W3, W4, W5, and W6 for Wagyu, and H3, H4, H5, and H6 for Holstein calves, respectively. This terminology was used solely to reflect the age-specific sampling points, not to imply grouping or separate management during the trial. All experimental procedures were approved by the Institutional Animal Care and Use Committee (IACUC) of Animal Welfare Committee of Heilongjiang Academy of Agricultural Sciences (Approval No. IHA20240520).

### Sample collection and detection

On days 1 (corresponding to 3 months of age), 45 (4 months), 75 (5 months), and 105 (6 months) of the formal trial period, rumen fluid samples were collected from all calves using an oral rumen tube prior to the morning feeding. To minimize contamination, the initial 200 mL of rumen fluid was discarded. Subsequent rumen fluid was collected, filtered through four layers of sterile gauze, and immediately snap-frozen in liquid nitrogen for subsequent analysis. Body weight on the day of collection are presented in Supplementary Table 1.

### DNA library construction and sequencing

The metagenome library construction and sequencing were conducted by Beijing Biomarker Technologies Co., Ltd. (Beijing, China). The DNA was automatically extracted and purified with the TGuide S96 Magnetic Soil/Stool DNA Kit (Tiangen Biotech Beijing Co., Ltd., Cat. No. DP812). The concentration and integrity of the extracted DNA were assessed using a nucleic acid quantification instrument (Thermo Fisher Scientific, Model NANODROP 2000) and agarose gel electrophoresis (Tiangen Biotech Beijing Co., Ltd., Cat. No. DP219), respectively. Qualified DNA samples were then used for library construction with VAHTS Universal Plus DNA Library Prep Kit for Illumina (Nanjing Vazyme Biotech Co., Ltd., Cat. No. ND61702). This process involved enzymatic DNA fragmentation, end-repair of fragmented DNA, 3’ adenylation, ligation of sequencing adapters, purification and size selection of ligated products, PCR amplification, and final purification. The concentration of the constructed libraries was measured using the Qubit 3.0 Fluorometer. Qualified libraries were then sequenced on the Illumina NovaSeq 6000 platform (PE 150). All experimental steps were performed according to the instruction manual.

### Bioinformatics analysis

#### Raw data processing

Raw sequencing data were filtered using Fastp software (v0.23.2, parameters: -5 -W 50 -M 20 -l 60 -n 0 -g -A) to remove reads with low-quality scores (Q < 20), ambiguous bases, or lengths less than 50 bp, thereby obtaining high-quality data (Clean Tags). Subsequently, host-derived sequences were identified and removed by aligning the reads to the bovine reference genome (ARS-UCD1.2) using Bowtie2 software (v2.2.4, parameters: --seed 123456 -I 200 -X 1000 --un-conc-gz). Clean, high-quality reads free of host contamination were used for downstream analyses.

#### Metagenome assembly and annotation

Metagenome assembly was performed with MEGAHIT (v1.1.2) using a k-mer range of 21–127 to ensure efficient assembly of the clean reads into contigs, and contigs shorter than 300 bp were filtered out. The assembly results were evaluated using QUAST software (v2.3, default parameters). Open reading frames (ORFs) were predicted from the assembled contigs using MetaGeneMark (v3.26, default parameters) to generate the initial gene catalog, and non-redundant gene sets were subsequently constructed using MMseqs2 (v12-113e3; 95% identity, 90% coverage).

The non-redundant gene sequences were then annotated by BLAST alignment (v0.9.29, alignment E-value threshold: 1e-5) against the NCBI Nr and KEGG databases, respectively. The microbial taxa from phylum to genus levels was subsequently inferred from NR databases, while the functional potential was predicted from KEGG databases.

#### Diversity analysis and differential testing

Alpha diversity metrics (Shannon, Simpson, ACE, and Chao1) were calculated using QIIME2 (v2023.5) to assess microbial richness and diversity within samples. Cross-sectional comparisons were analyzed using one-way ANOVA, followed by Dunn’s post-hoc test for pairwise comparisons, and longitudinal comparisons were assessed using independent samples t-tests. Effect sizes were estimated using eta squared (*η*^*2*^, The value of low effect size, moderate effect size and high effect size was 0.01, 0.06, 0.14) and Cohen’s d (|*d*|, The value of low effect size, moderate effect size and high effect size was 0.20, 0.50, 0.80), respectively. Beta diversity was assessed using Principal Coordinate Analysis (PCoA) based on the Bray-Curtis dissimilarity matrix, and intergroup differences were tested using Permutational Multivariate Analysis of Variance (PERMANOVA) performed with the vegan package (v2.6-4) in R (v4.3.0).

#### Linear discriminant analysis (LDA)-Based differential taxonomic

Linear discriminant analysis effect size (LEfSe, LDA ≥ 2.0) testing was performed in order to identify differentially abundant bacterial taxa from phylum to genus level. Multiple-testing correction was performed using the Benjamini-Hochberg false discovery rate (FDR < 0.05). Specifically, we used the Benjamini-Hochberg adjusted *P*-values (q-value < 0.05).

#### Functional pathway analysis of metagenome

Given that most functional pathway data were not normally distributed, nonparametric statistics were employed: Kruskal-Wallis test for longitudinal levels of functional pathway, and Mann-Whitney U test for cross-sectional levels. Dunn’s post hoc tests were carried out with adjustments using Bonferroni correction. Effect sizes were estimated using conversion value of eta squared (*η*^*2*^, conversion value=(H-k + 1)/(n-k)) and Cliff’s delta (|*δ*|, The value of low effect size, moderate effect size and high effect size was 0.147, 0.33, 0.474), respectively.

### Data processing

We assessed normality and homogeneity of variances for all continuous variables. Alpha-diversity indices met both assumptions, whereas functional-pathway gene abundances only partially conformed and were therefore analyzed with non-parametric tests. These analyses and visualizations were performed using R (v3.1.1) and Python (v2.0), respectively. A *P*-value < 0.05 was considered statistically significant.

## Results

### Composition of rumen microbiota

#### Phylum-Level distribution of rumen microbiota

**Fig. 1:**
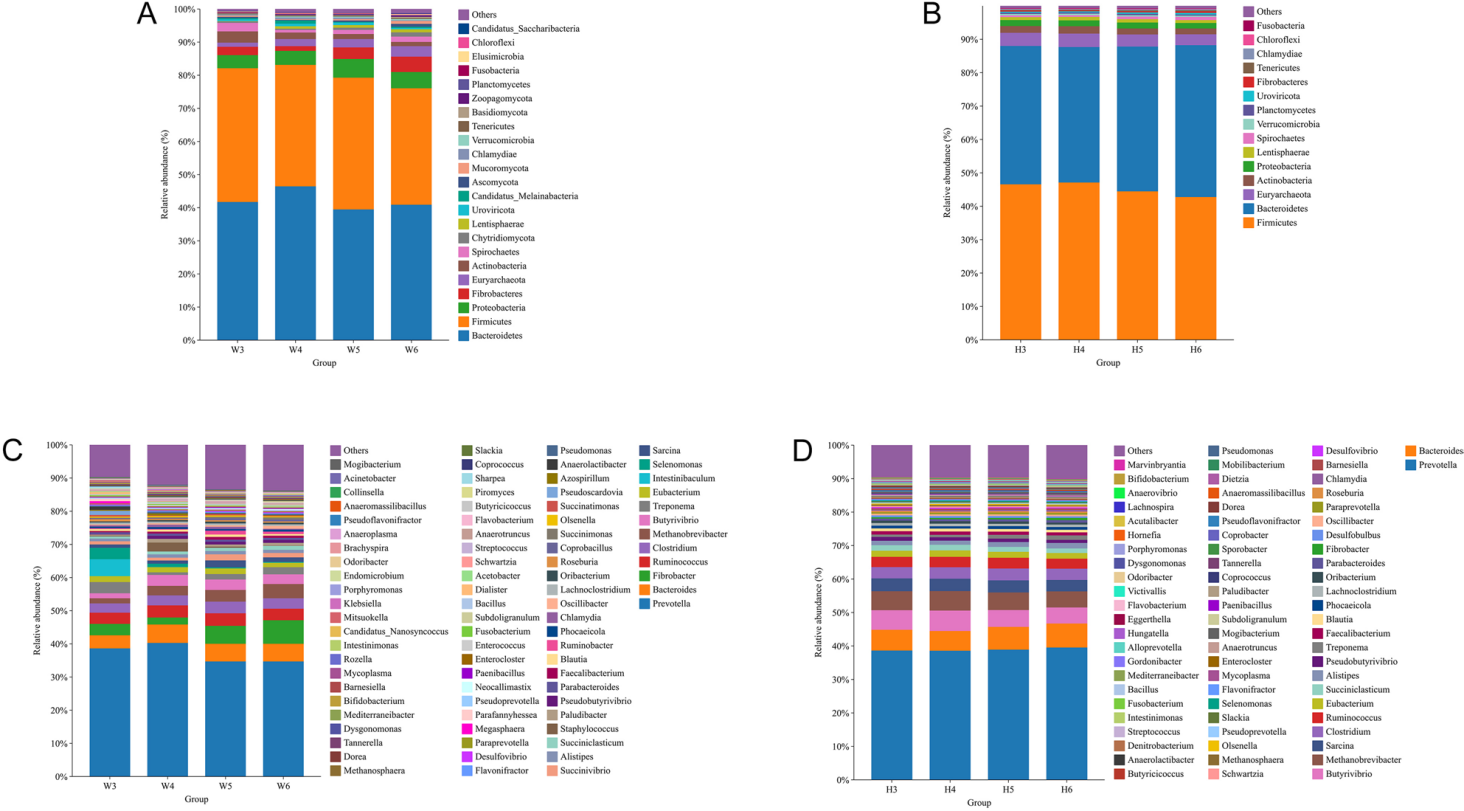
Rumen microbiota composition in Wagyu and Holstein calves. Phylum-level relative abundance ( >0.1%) in Wagyu (**A**, n=5) and Holstein (**B**, n=5) calves at 3, 4, 5 and 6 months of age. Genus-level relative abundance ( >0.1%) in Wagyu (**C**, n=5) and Holstein (**D**, n=5) calves at 3, 4, 5 and 6 months of age

Based on metagenomic annotations with a relative abundance threshold of > 0.1%, selected as a balanced criterion to ensure sensitivity in detecting both dominant and rare taxa while avoiding overestimation of low-confidence annotations, a total of 23 phyla were identified in the rumen microbiota of Wagyu calves and 15 phyla in Holstein calves during the calf stage (Fig. 1A and B). At the phylum level, the combined proportion of the top five taxa in 3-month-old Wagyu calves was 92.03% (Bacteroidetes 41.75%, Firmicutes 40.39%, Proteobacteria 3.96%, Actinobacteria 3.36%, Fibrobacteres 2.58%), by 6 months it fell to 88.78%, with Fibrobacteres (4.63%) and Euryarchaeota (3.15%) replacing Actinobacteria (1.29%) and Spirochaetes (1.64%) in the top five. In Holstein calves, the corresponding values were 95.77% at 3 months (Firmicutes 46.59%, Bacteroidetes 44.14%, Euryarchaeota 3.92%, Actinobacteria 2.03%, Proteobacteria 1.79%) and 94.89% at 6 months, with Bacteroidetes (45.45%) overtaking Firmicutes (42.79%) (Table S2).

In Wagyu calves, the dominant phyla included Firmicutes and Bacteroidetes, which exhibited dynamic fluctuations over time. The relative abundance of Firmicutes decreased from 40.39% at 3 months to 36.70% at 4 months, followed by a transient increase to 39.81% at 5 months, and a subsequent decline to 35.18% at 6 months. Bacteroidetes remained relatively stable, fluctuating between 39.48% and 46.45%. Additionally, Proteobacteria, Fibrobacteres, and Euryarchaeota showed gradual increases from 3 to 6 months of age, suggesting age-related shifts in rumen fermentation-related microbial populations. (Fig. 1A)

In Holstein calves, Firmicutes consistently dominated, with a slight decrease from 47.13% at 4 months to 42.79% at 6 months, while Bacteroidetes increased from 40.58% to 45.45% over the same period, indicating a gradual adjustment toward a Bacteroidetes-enriched profile with age. Euryarchaeota, a key archaeal phylum associated with methanogenesis, showed a slight decrease from 4.00% at 4 months to 3.27% at 6 months. Minor phyla such as Actinobacteria and Proteobacteria remained relatively stable at low abundance throughout the study period. (Fig. 1B)

Between breeds, differences were observed at the phylum level. At each age point, Holstein calves exhibited consistently higher proportions of Firmicutes and Euryarchaeota compared to Wagyu calves, whereas Wagyu calves displayed higher relative abundances of Fibrobacteres and Proteobacteria, particularly evident from 5 months of age onward (Fig. 1A and B).

#### Rumen microbial composition at the genus level

At the genus level, based on metagenomic annotations with a relative abundance threshold of > 0.1%—a threshold selected to balance sensitivity and specificity—a total of 83 genera were identified in the rumen microbiota of Wagyu calves and 73 genera in Holstein calves during the calf stage (Fig. 1C and D). Among these, 57 genera were common to both groups, with 26 unique to Wagyu and 16 unique to Holstein. At the genus level, the cumulative relative abundance of the ten most abundant genera declined from 67.45% to 65.82% in Wagyu and from 71.45% to 70.82% in Holstein over the same period (Table S3).

In Wagyu calves, *Prevotella* dominated across all time points, although its relative abundance gradually declined from 38.63% at 3 months to 34.69% at 6 months, indicating a progressive diversification of other genera with calf maturation. Alongside *Prevotella*, *Fibrobacter* increased from 3.39% at 3 months to 7.07% at 6 months, suggesting enhanced fiber degradation capacity with age. *Methanobrevibacter*, a methanogen genus, also increased notably from 1.54% to 4.29%, paralleling the development of methane-producing communities. Conversely, genera such as *Intestinibaculum* and *Treponema* showed early dominance but decreased substantially by 6 months of age, reflecting a shift toward a more mature and stable microbial profile. (Fig. 1C)

In Holstein calves, *Prevotella* remained relatively stable at around 38.56–39.54%, with minimal fluctuations over time. Notably, *Butyrivibrio*, *Methanobrevibacter*, and *Bacteroides* maintained consistently higher proportions compared to Wagyu calves throughout the study, with *Methanobrevibacter* exhibiting a slight decrease from 5.81% at 4 months to 4.80% at 6 months, indicating possible breed-specific dynamics in methanogenic communities. Additionally, *Sarcina*, a genus associated with carbohydrate fermentation, showed stable abundance across all time points, while *Succiniclasticum* and *Alistipes* remained unique contributors to Holstein rumen microbiota but were less prominent in Wagyu calves. (Fig. 1D)

Between breeds, key differences emerged at the genus level. Holstein calves exhibited consistently higher levels of *Methanobrevibacter*, *Butyrivibrio*, and *Succiniclasticum*, indicating a potential preference for hydrogen utilization and butyrate production pathways. In contrast, Wagyu calves displayed higher proportions of *Fibrobacter* and *Treponema*. (Figure 1C and D)

### LEfSe analysis of rumen microbial composition

#### Longitudinal LEfSe analysis of rumen microbial composition

**Fig. 2:**
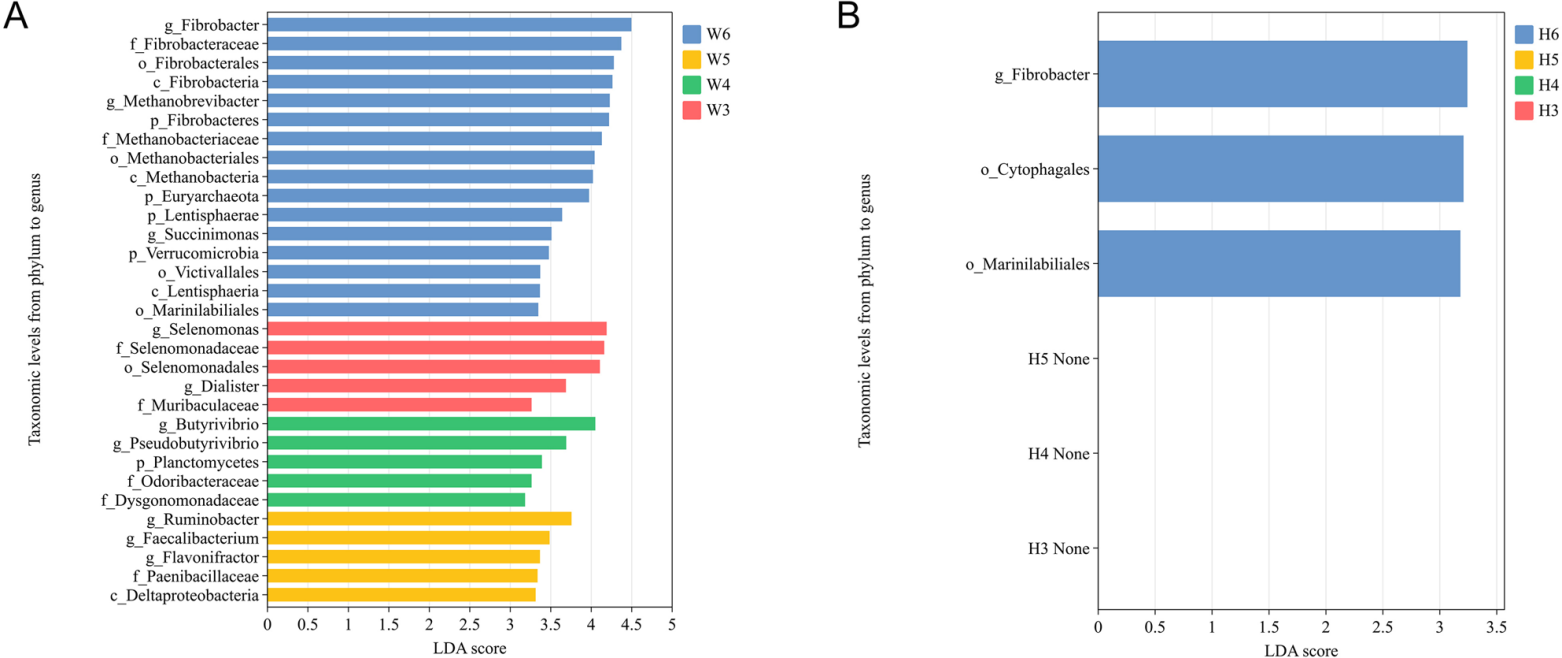
LEfSe analysis (LDA score ≥ 2.0, q < 0.05) of longitudinal comparison of rumen microbiota in Wagyu (**A**, n=5) and Holstein (**B**, n=5) from 3 to 6 months of age

To explore age-associated shifts in rumen microbiota within each breed, LEfSe analysis (LDA score ≥ 2.0, FDR < 0.05) was performed at taxonomic levels from phylum to genus, using other developmental stages within the same breed as the reference group for each time point (Fig. 2A and B).

In Wagyu calves (Fig. 2A), distinct microbial biomarkers were identified at each developmental stage. At 3 months, taxa such as *Selenomonas* and its affiliated family Selenomonadaceae, as well as *Dialister*, were significantly enriched compared to other time points, indicating their potential roles in early rumen development. At 4 months, genera associated with butyrate production, including *Butyrivibrio* and *Pseudobutyrivibrio*, became significantly enriched, reflecting shifts toward fibrolytic and saccharolytic capacities. By 5 months, an enrichment of Ruminobacter and Faecalibacterium suggested enhanced propionate production and anti-inflammatory microbial functions. At 6 months, a broader diversification was observed, with *Fibrobacter*, *Methanobrevibacter*, and their corresponding higher taxonomic ranks (*Fibrobacteraceae*, *Fibrobacterales*, etc.) showing significant enrichment, indicative of a maturing fibrolytic and methanogenic community structure.

In contrast, Holstein calves (Fig. 2B) exhibited fewer dynamic shifts across the developmental period. Significant differences were detected exclusively at 6 months, with *Fibrobacter*, as well as members of *Cytophagales* and *Marinilabiliales*, enriched relative to earlier stages (q < 0.05).

#### Cross-sectional LEfSe analysis of rumen microbial composition

**Fig. 3:**
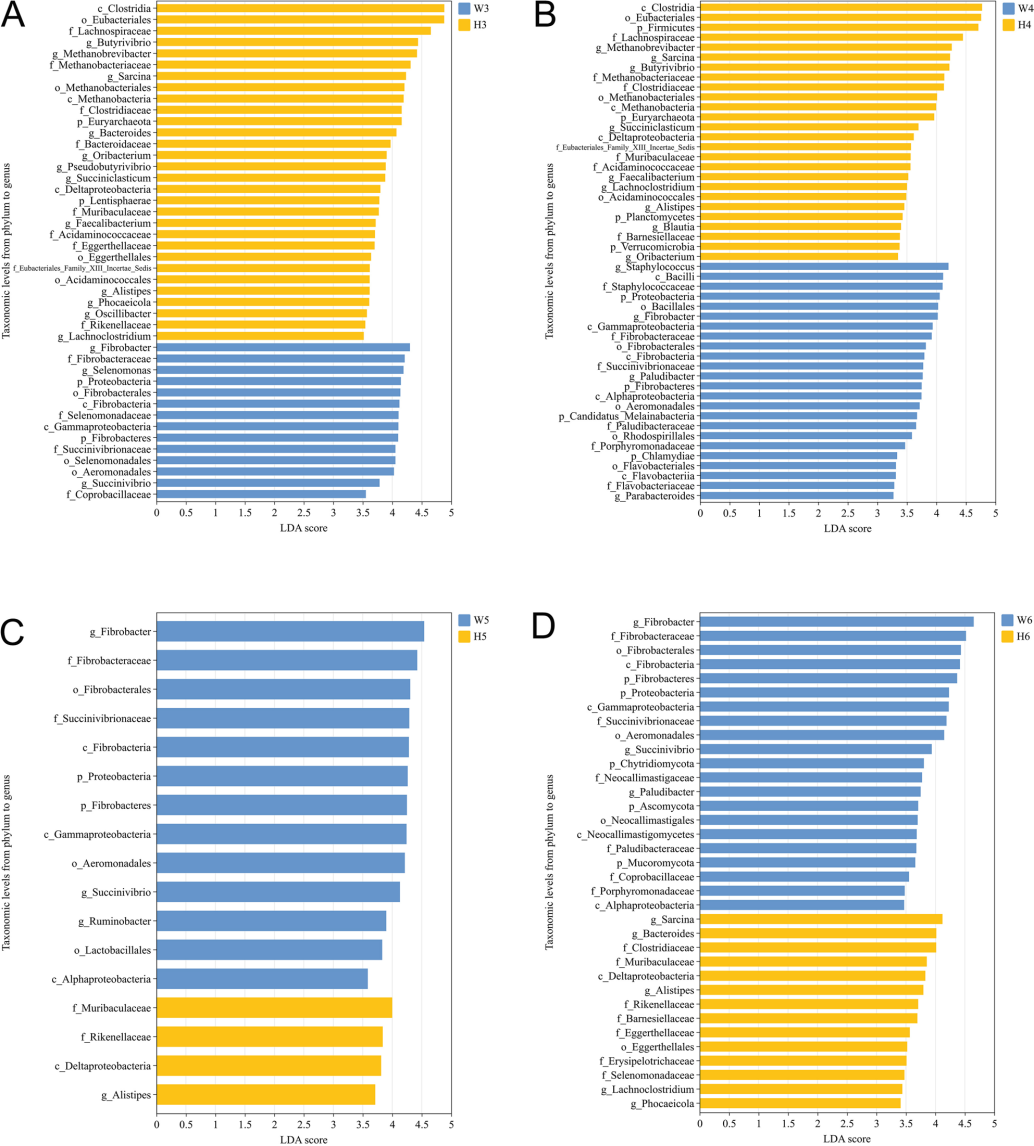
LEfSe analysis (LDA score ≥ 2.0, q < 0.05) of cross-sectional comparison of rumen microbiota between Wagyu (*n* = 5) and Holstein (*n* = 5) calves at 3 (**A**), 4 (**B**), 5 (**C**), and 6 (**D**) months of age

To identify breed-specific rumen microbial biomarkers at each developmental stage, LEfSe analysis was performed comparing Wagyu and Holstein calves at the same age, with each breed serving as the reference group for the other (Fig. 3A-D).

At 3 months of age (Fig. 3A), Wagyu calves exhibited enrichment of taxa including *Fibrobacter*, *Selenomonas*, and *Proteobacteria*, suggesting early establishment of fibrolytic and saccharolytic functions. Conversely, Holstein calves showed a predominance of taxa such as *Clostridia*, *Eubacteriales*, and *Lachnospiraceae*, indicating a gut microbiota more oriented toward butyrate-producing *Firmicutes* and *Methanobrevibacter*.

At 4 months of age (Fig. 3B), Wagyu calves maintained higher abundances of *Staphylococcus*, *Bacilli*, and *Proteobacteria*, while Holstein calves continued to exhibit an enrichment of Clostridia, Eubacteriales, Firmicutes, and *Methanobrevibacter.*

At 5 months of age (Fig. 3C), the divergence became more pronounced. Wagyu calves demonstrated significant enrichment of *Fibrobacter* and *Succinivibrionaceae*, associated with cellulolytic and propionate-producing capacities, whereas Holstein calves displayed a more restricted set of differential taxa, including Muribaculaceae, Rikenellaceae, and Alistipes, indicative of a less diverse fibrolytic community.

At 6 months of age (Fig. 3D), Wagyu calves continued to harbor a richer diversity of fibrolytic taxa, including *Fibrobacter*, Fibrobacteraceae, and Fibrobacterales, while Holstein calves were characterized by higher abundances of *Sarcina*, *Bacteroides*, and Muribaculaceae, suggesting breed-related differences in fermentation capacity and fiber degradation efficiency persisting into later developmental stages.

These cross-sectional findings emphasize distinct microbial compositional trajectories between breeds (q < 0.05), with Wagyu calves exhibiting earlier and more diverse fibrolytic microbial establishment, while Holstein calves maintained a community dominated by butyrate-producing Firmicutes and methanogens across developmental stages.

### Rumen microbial diversity

#### Alpha diversity of rumen microbiota

Based on the premise that the early postnatal period represents a critical window for rumen microbial colonization and functional differentiation, this study employed alpha diversity metrics—including the Shannon index, Simpson index, ACE index, and Chao1 index—to evaluate the succession and maturation trajectories of rumen microbial communities. These indices were used to assess how breed (Wagyu vs. Holstein) and age influence community stability and compositional complexity during calf development.

As presented in Table 3, within-breed comparisons revealed that age significantly affected microbial diversity in Wagyu calves, with Shannon, ACE, and Chao1 indices at 5 and 6 months of age significantly higher than those at 3 months of age (*P* < 0.05). In contrast, no statistically significant age-related changes in alpha diversity were observed in Holstein calves, suggesting a delayed or more stable colonization pattern in this breed. In between-breed comparisons, alpha diversity indices did not significantly differ at 3 months of age. However, from 4 months onward, Wagyu calves exhibited higher richness indices (ACE and Chao1) than Holstein calves, with these differences reaching statistical significance at 4 and 5 months (*P* < 0.05). By 6 months, all three indices (Shannon, ACE, and Chao1) were significantly elevated in Wagyu calves compared to Holstein calves (*P* < 0.05), which may be indicating enhanced microbial complexity and potential functional maturation in this breed.

**Table 3:** Alpha diversity of rumen microbiota

Items	Breed Month old	3	4	5	6	SEM	*P*-Value	η²-Value
Shannon index	Wagyu	3.17b	3.40ab	3.63a	3.64a*	0.07	0.05	0.41
	Holstein	3.28	3.29	3.29	3.29	0.04	1.00	< 0.01
	SEM	0.10	0.08	0.09	0.07	-	-	-
	*P*-Value	0.60	0.52	0.05	< 0.01	-	-	-
	*|d|-Value*	0.31	0.24	0.21	0.10	-	-	-
Simpson index	Wagyu	0.81	0.82	0.86	0.86	0.01	0.25	0.23
	Holstein	0.83	0.83	0.83	0.83	0.01	1.00	< 0.01
	SEM	0.02	0.01	0.01	0.01	-	-	-
	*P*-Value	0.61	0.8	0.20	0.07	-	-	-
	*|d|-Value*	0.06	0.04	0.03	0.03	-	-	-
ACE index	Wagyu	2099.41b	2449.19ab*	2660.64a*	2698.78a*	84.02	0.03	0.45
	Holstein	2188.81	2189.33	2220.82	2247.07	12.41	0.30	0.20
	SEM	114.23	58.30	81.93	76.79	-	-	-
	*P*-Value	0.72	0.01	< 0.01	< 0.01	-	-	-
	*|d|-Value*	379.86	130.92	87.36	50.65	-	-	-
Chao l index	Wagyu	2096.87b	2446.69ab*	2658.49a*	2696.02a*	84.08	0.03	0.45
	Holstein	2184.08	2184.76	2215.96	2242.69	12.44	0.30	0.20
	SEM	114.27	58.66	82.40	77.09	-	-	-
	*P*-Value	0.73	0.01	< 0.01	< 0.01	-	-	-
	*|d|-Value*	380.16	131.44	87.59	51.27	-	-	-

Values within the same row sharing no letters or identical letters (e.g., a, b) indicate no significant difference (*P* > 0.05), while different lowercase letters indicate significant differences (*P* < 0.05) based on longitudinal comparisons within the same breed across ages (3, 4, 5, and 6 months). Asterisks indicate a significant difference (*P* < 0.05) in cross-sectional comparisons between Wagyu and Holstein calves at the same age. SEM refers to standard error of the mean. The same annotation criteria apply to the following tables

#### Beta diversity of rumen microbiota

**Fig. 4:**
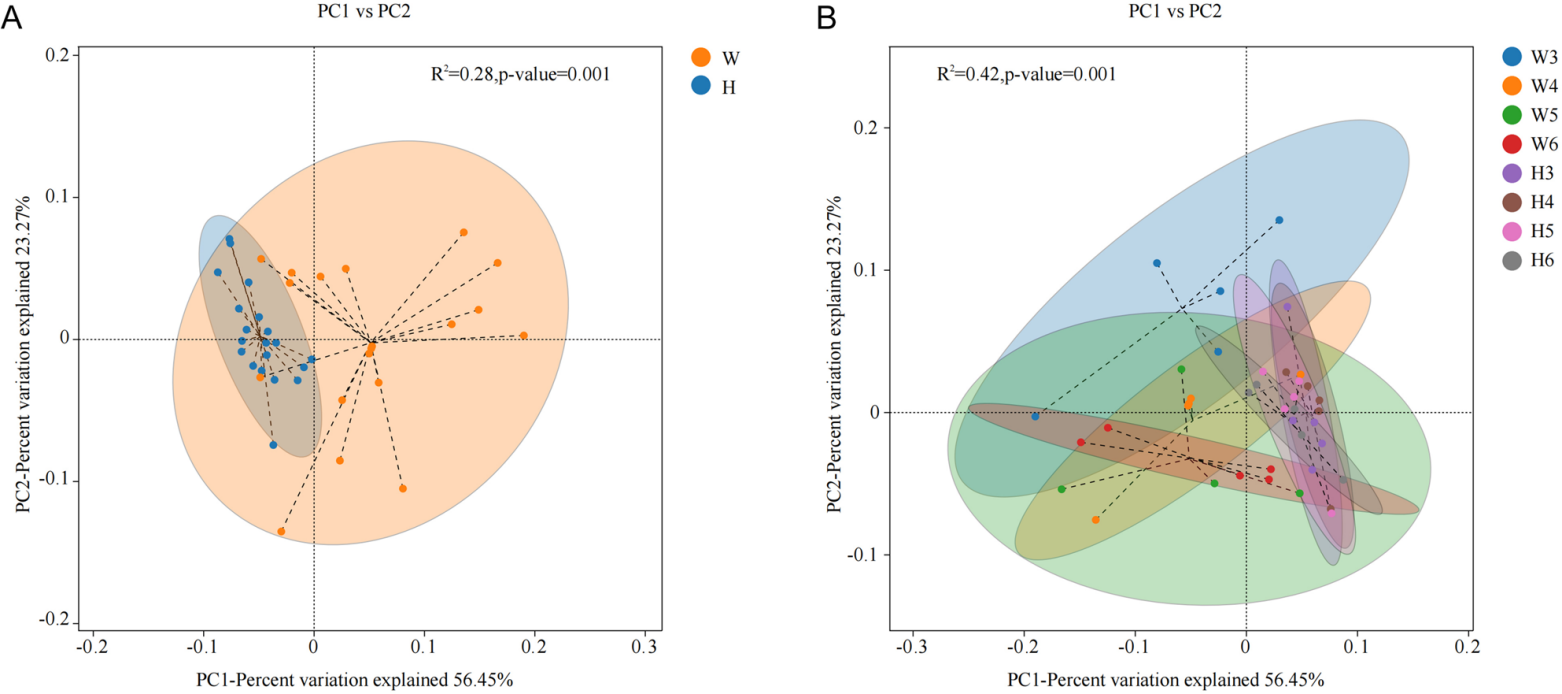
PCoA (Bray-Curtis dissimilarity, ellipses 95% CI) of rumen microbiota diversity in Wagyu (**A**, *n* = 5) and Holstein (**B**, *n* = 5) calves

As illustrated in Fig. 4, principal coordinate analysis (PCoA) based on the Bray-Curtis dissimilarity matrix at the genus level was conducted to assess beta diversity in the rumen microbiota. Figure 4A indicates a clear separation between Wagyu and Holstein calves, indicating significant interbreed differences in microbial community composition. Notably, the microbial communities in Wagyu calves displayed greater dispersion, suggesting lower inter-individual similarity and higher variability compared to Holstein calves. Figure 4B shows dynamic temporal changes in microbial similarity across developmental stages. The degree of variation in microbial community structure over time was more pronounced in Wagyu calves.

### Functional pathway characteristics of rumen microbiota

#### KEGG functional pathway annotation of rumen microbiota

**Fig. 5:**
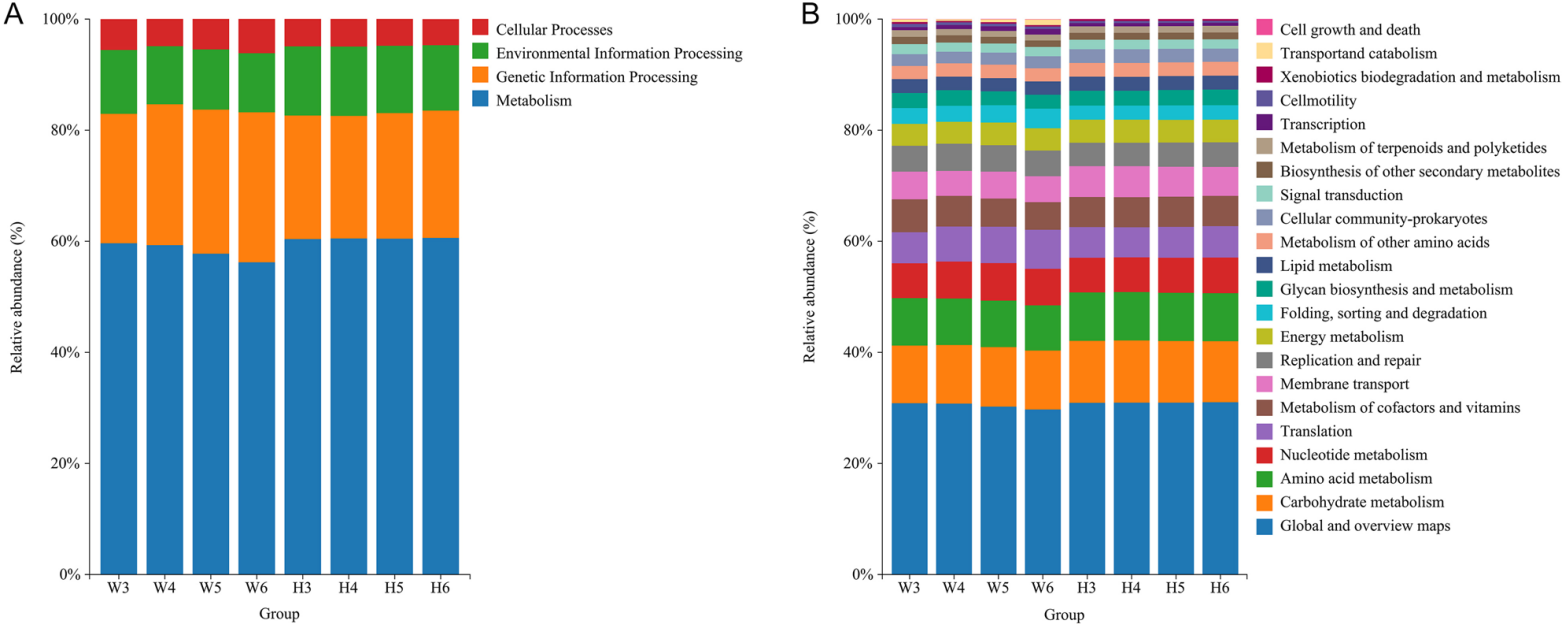
KEGG functional pathway annotation of rumen microbiota at Level 1 (**A**) and Level 2 (**B**) in Wagyu (*n* = 5) and Holstein (*n* = 5) calves

As shown in Fig. 5, functional annotation of rumen microbial genes using the KEGG database identified 4 level 1 and 22 level 2 functional pathways in both Wagyu and Holstein calves. At the level 1 classification (Fig. 5A), the overall distribution of functional gene categories was comparable between breeds. The predominant pathways, in descending order of relative abundances, were Metabolism, Genetic Information Processing, Environmental Information Processing, and Cellular Processes. At the level 2 classification (Fig. 5B), the top ten functional pathways included: Global and Overview Maps, Carbohydrate Metabolism, Amino Acid Metabolism, Nucleotide Metabolism, Metabolism of Cofactors and Vitamins, Translation, Membrane Transport, Replication and Repair, Energy Metabolism, and Folding, Sorting and Degradation. Notably, the tenth-ranked pathway differed between breeds: Glycan Biosynthesis and Metabolism in Holstein calves instead of Folding, Sorting and Degradation as in Wagyu calves, possibly suggesting subtle breed-dependent variations in microbial functional potential.

#### Comparison of KEGG functional pathway in rumen microbiota

As presented in Table 4, both age and breed influenced the functional gene composition of rumen microbial in calves. At the level 1 classification, age-related changes were observed only in Wagyu calves. By 6 months, the relative abundance of Metabolism-related genes in Wagyu calves was significantly lower than that at 3 and 4 months (*P* < 0.05), while Cellular Processes-related genes was significantly higher than that at 4 months (*P* < 0.05). No significant age-related differences were observed in Holstein calves.

**Table 4:** Comparison of KEGG pathway gene abundance in rumen microbiota

Items	Breed Month old	3	4	5	6	SEM	*P*-Value	η²-Value
Level 1								
Metabolism	Wagyu	59.64a	59.30a	57.77ab	56.22b	0.48	0.04	0.37
	Holstein	60.39	60.50	60.45*	60.58*	0.10	0.78	0
	SEM	0.55	0.36	0.56	0.78	-	-	-
	*P*-Value	0.60	0.12	0.01	0.01	-	-	-
	|*δ*|-Value	1.00	0.60	1.00	1.00	-	-	-
Genetic Information Processing	Wagyu	23.32	25.37*	25.95*	27.02*	0.56	0.11	0.20
	Holstein	22.26	22.07	22.66	22.97	0.14	0.08	0.24
	SEM	0.60	0.73	0.73	0.75	-	-	-
	*P*-Value	0.60	0.01	0.01	0.01	-	-	-
	|*δ*|-Value	1.00	1.00	1.00	1.00	-	-	-
Environmental Information Processing	Wagyu	11.50	10.46	10.82	10.63	0.26	0.85	0
	Holstein	12.44	12.48*	12.08*	11.76	0.14	0.26	0.06
	SEM	0.40	0.42	0.32	0.30	-	-	-
	*P*-Value	0.18	0.01	0.03	0.08	-	-	-
	|*δ*|-Value	0.68	1.00	0.90	0.68	-	-	-
Cellular Processes	Wagyu	5.54ab	4.88b	5.46ab	6.14a*	0.18	0.03	0.42
	Holstein	4.91	4.95	4.81	4.69	0.05	0.23	0.08
	SEM	0.20	0.06	0.14	0.32	-	-	-
	*P*-Value	0.08	0.60	0.05	0.01	-	-	-
	|*δ*|-Value	0.52	0.20	0.80	1.00	-	-	-
Level 2								
Global and overview maps	Wagyu	30.85a	30.76a	30.22ab*	29.71b	0.14	0.01	0.56
	Holstein	30.90	30.94	30.94	31.00*	0.04	0.90	0.15
	SEM	0.18	0.08	0.14	0.23	-	-	-
	*P*-Value	0.75	0.25	0.01	0.01	-	-	-
	|*δ*|-Value	0.12	0.44	1.00	1.00	-	-	-
Carbohydrate metabolism	Wagyu	10.38	10.57	10.74	10.64	0.11	0.81	0.13
	Holstein	11.19*	11.23*	11.13	11.03	0.05	0.51	0.04
	SEM	0.17	0.17	0.14	0.13	-	-	-
	*P*-Value	0.01	0.04	0.33	0.18	-	-	-
	|*δ*|-Value	1.00	0.76	0.40	0.52	-	-	1
Amino acid metabolism	Wagyu	8.53	8.37	8.37	8.10	0.09	0.38	0.01
	Holstein	8.71	8.71*	8.66	8.64*	0.02	0.35	0.02
	SEM	0.14	0.08	0.10	0.12	-	-	-
	*P*-Value	0.60	0.01	0.09	0.01	-	-	-
	|*δ*|-Value	0.20	1.00	0.70	1.00	-	-	-
Nucleotide metabolism	Wagyu	6.27	6.65*	6.77*	6.58	0.09	0.28	0.05
	Holstein	6.25b	6.22b	6.30ab	6.41a	0.02	0.01	0.49
	SEM	0.15	0.12	0.12	0.05	-	-	-
	*P*-Value	0.92	0.02	0.01	0.08	-	-	-
	|*δ*|-Value	0.04	0.92	1.00	0.68	-	-	-
Translation	Wagyu	5.62b	6.30ab*	6.53ab*	7.06a*	0.18	0.04	0.37
	Holstein	5.50	5.41	5.57	5.66	0.04	0.12	0.18
	SEM	0.24	0.22	0.22	0.25	-	-	-
	*P*-Value	0.35	0.01	0.01	0.01	-	-	-
	|*δ*|-Value	0.36	1.00	1.00	1.00	-	-	-
Metabolism of cofactors and vitamins	Wagyu	5.92a*	5.51ab	5.09bc	4.95c	0.11	0.01	0.58
	Holstein	5.42	5.44	5.44	5.42*	0.03	0.93	0.16
	SEM	0.17	0.09	0.10	0.09	-	-	-
	*P*-Value	0.04	0.75	0.14	0.01	-	-	-
	|*δ*|-Value	0.76	0.12	0.60	1.00	-	-	-
Membrane transport	Wagyu	4.98	4.50	4.81	4.68	0.14	0.75	0.12
	Holstein	5.56	5.58*	5.39	5.23	0.08	0.33	0.03
	SEM	0.22	0.22	0.16	0.16	-	-	-
	*P*-Value	0.18	0.01	0.05	0.18	-	-	-
	|*δ*|-Value	0.52	1.00	0.80	0.52	-	-	-
Replication and repair	Wagyu	4.66	4.89*	4.80*	4.61	0.10	0.67	0.10
	Holstein	4.23	4.22	4.34	4.43	0.03	0.10	0.21
	SEM	0.17	0.15	0.13	0.08	-	-	-
	*P*-Value	0.60	0.02	0.03	0.35	-	-	-
	|*δ*|-Value	0.20	0.92	0.90	0.36	-	-	-
Energy metabolism	Wagyu	3.93	3.96	4.07	4.02	0.05	0.68	0.10
	Holstein	4.13	4.13	4.08	4.06	0.02	0.46	0.03
	SEM	0.07	0.05	0.04	0.06	-	-	-
	*P*-Value	0.12	0.12	0.81	0.75	-	-	-
	|*δ*|-Value	0.60	0.60	0.10	0.12	-	-	-
Folding, sorting and degradation	Wagyu	2.84	2.89*	3.13*	3.54*	0.11	0.08	0.25
	Holstein	2.56	2.56	2.61	2.60	0.01	0.15	0.15
	SEM	0.13	0.08	0.10	0.19	-	-	-
	*P*-Value	0.92	0.01	0.01	0.01	-	-	-
	|*δ*|-Value	0.04	1.00	1.00	1.00	-	-	-
Glycan biosynthesis and metabolism	Wagyu	2.73	2.78	2.51	2.48	0.05	0.09	0.24
	Holstein	2.68	2.68	2.76	2.81*	0.03	0.42	0.01
	SEM	0.08	0.04	0.07	0.07	-	-	-
	*P*-Value	0.47	0.12	0.05	0.02	-	-	-
	|*δ*|-Value	0.28	0.60	0.80	0.92	-	-	-
Lipid metabolism	Wagyu	2.49	2.43	2.36	2.40	0.02	0.09	0.23
	Holstein	2.50	2.51*	2.53*	2.53	0.01	0.82	0.13
	SEM	0.03	0.02	0.04	0.04	-	-	-
	*P*-Value	0.75	0.02	0.01	0.08	-	-	-
	|*δ*|-Value	0.12	0.92	1.00	0.68	-	-	-
Metabolism of other amino acids	Wagyu	2.37	2.39	2.41	2.39	0.02	0.98	0.19
	Holstein	2.48	2.48*	2.46	2.49*	0.01	0.44	0.02
	SEM	0.04	0.02	0.03	0.02	-	-	-
	*P*-Value	0.47	0.01	0.46	0.01	-	-	-
	|*δ*|-Value	0.28	1.00	0.30	1.00	-	-	-
Cellular community - prokaryotes	Wagyu	2.11	2.09	2.19	2.13	0.05	0.81	0.14
	Holstein	2.45	2.47*	2.42*	2.37	0.03	0.58	0.07
	SEM	0.08	0.08	0.06	0.07	-	-	-
	*P*-Value	0.12	0.01	0.03	0.12	-	-	-
	|*δ*|-Value	0.60	1.00	0.90	0.60	-	-	-
Signal transduction	Wagyu	1.82	1.67	1.62	1.70	0.03	0.09	0.24
	Holstein	1.73	1.73	1.69	1.67	0.01	0.06	0.28
	SEM	0.05	0.03	0.03	0.02	-	-	-
	*P*-Value	0.12	0.47	0.33	0.92	-	-	-
	|*δ*|-Value	0.60	0.28	0.40	0.04	-	-	-
Biosynthesis of other secondary metabolites	Wagyu	1.30a	1.28a*	1.20ab	1.17b	0.02	0.01	0.56
	Holstein	1.22	1.21	1.22	1.22*	< 0.01	0.49	0.04
	SEM	0.03	0.01	0.02	0.01	-	-	-
	*P*-Value	0.12	0.01	0.33	0.01	-	-	-
	|*δ*|-Value	0.60	1.00	0.40	1.00	-	-	-
Metabolism of terpenoids and polyketides	Wagyu	1.20a	1.16a	1.06b	1.05b	0.02	0.01	0.66
	Holstein	1.17	1.17	1.17*	1.19*	0.01	0.64	0.08
	SEM	0.03	0.01	0.03	0.02	-	-	-
	*P*-Value	0.18	0.60	0.03	0.01	-	-	-
	|*δ*|-Value	0.52	0.20	0.90	1.00	-	-	-
Transcription	Wagyu	0.56b	0.72b	0.85ab*	0.98a*	0.05	0.02	0.45
	Holstein	0.58	0.57	0.58	0.58	< 0.01	0.59	0.07
	SEM	0.07	0.04	0.06	0.07	-	-	-
	*P*-Value	0.60	0.12	0.01	0.01	-	-	-
	|*δ*|-Value	0.20	0.60	1.00	1.00	-	-	-
Cell motility	Wagyu	0.60	0.45	0.47	0.45*	0.04	0.46	0.03
	Holstein	0.38	0.39	0.35	0.33	0.01	0.23	0.08
	SEM	0.07	0.04	0.06	0.03	-	-	-
	*P*-Value	0.12	0.35	0.46	0.01	-	-	-
	|*δ*|-Value	0.60	0.36	0.30	1.00	-	-	-
Xenobiotics biodegradation and metabolism	Wagyu	0.31	0.30	0.29	0.28	0.01	0.83	0.14
	Holstein	0.34	0.34*	0.33*	0.31	0.01	0.14	0.16
	SEM	0.02	0.01	0.01	0.01	-	-	-
	*P*-Value	0.12	0.05	0.01	0.35	-	-	-
	|*δ*|-Value	0.60	0.76	1.00	0.36	-	-	-
Transport and catabolism	Wagyu	0.49	0.28*	0.50*	1.00*	0.13	0.26	0.07
	Holstein	< 0.01	< 0.01	0.01	0.01	< 0.01	0.34	0.02
	SEM	0.15	0.08	0.10	0.24	-	-	-
	*P*-Value	0.12	0.01	0.01	0.01	-	-	-
	|*δ*|-Value	0.60	1.00	1.00	1.00	-	-	-
Cell growth and death	Wagyu	0.03	0.02*	0.04*	0.06*	0.01	0.34	0.03
	Holstein	< 0.01	< 0.01	< 0.01	< 0.01	0	0.91	0.15
	SEM	0.01	0.01	0.01	0.01	-	-	-
	*P*-Value	0.14	0.01	0.01	0.01	-	-	-
	|*δ*|-Value	0.56	1.00	1.00	1.00	-	-	-

At the level 2 classification, Wagyu calves at 6 months exhibited significantly lower relative abundances of genes involved in Global and Overview Maps, Metabolism of Cofactors and Vitamins, Biosynthesis of Other Secondary Metabolites, and Transcription compared to 3 and 4 months (*P* < 0.05). In contrast, Translation-related gene abundance was significantly higher than that at 3 months (*P* < 0.05). Additionally, genes associated with Metabolism of Terpenoids and Polyketides were significantly less abundant at 5 and 6 months compared to 3 and 4 months (*P* < 0.05). Holstein calves exhibited no significant age-dependent changes in level 2 pathways.

Regarding interbreed comparisons, at 3 months, no significant differences in level 1 pathways were observed. At 4 months, Wagyu calves had higher abundances of Genetic Information Processing (*P* < 0.05) and lower abundances of Environmental Information Processing genes than Holstein calves (*P* < 0.05). These differences persisted and expanded at 5 and 6 months: Genetic Information Processing remained higher in Wagyu calves, while Metabolism and Environmental Information Processing were lower (*P* < 0.05). Additionally, at 6 months, Cellular Processes genes were more abundant in Wagyu than in Holstein calves (*P* < 0.05).

At the level 2 classification, breed-dependent functional differences became increasingly evident with age:

At 3 months of age, Wagyu calves showed higher abundance of genes for Metabolism of Cofactors and Vitamins (*P* < 0.05) but lower Carbohydrate Metabolism (*P* < 0.05).

At 4 months, Wagyu calves exhibited higher levels of Nucleotide Metabolism, Translation, Replication and Repair, Folding, Sorting and Degradation, Metabolism of Terpenoids and Polyketides, Transport and Catabolism, and Cell Growth and Death (*P* < 0.05). Conversely, Holstein calves had higher levels of Carbohydrate Metabolism, Amino Acid Metabolism, Membrane Transport, Lipid Metabolism, Metabolism of Other Amino Acids, Cellular Community–Prokaryotes, and Xenobiotics Biodegradation and Metabolism (*P* < 0.05).

At 5 months, similar trends persisted: Wagyu calves had higher relative abundances of Global and Overview Maps, Nucleotide Metabolism, Translation, Replication and Repair, Folding, Sorting and Degradation, Transcription, Transport and Catabolism, and Cell Growth and Death than Holstein calves (*P* < 0.05). In contrast, Holstein calves had higher levels of Lipid Metabolism, Cellular Community–Prokaryotes, Metabolism of Terpenoids and Polyketides, and Xenobiotics Biodegradation and Metabolism (*P* < 0.05).

At 6 months, Wagyu calves showed elevated abundances of genes involved in Translation, Folding, Sorting and Degradation, Transcription, Cell Motility, Transport and Catabolism, and Cell Growth and Death (*P* < 0.05). Whereas Holstein calves had higher abundances of Amino Acid Metabolism, Metabolism of Cofactors and Vitamins, Glycan Biosynthesis and Metabolism, Metabolism of Other Amino Acids, Biosynthesis of Other Secondary Metabolites, and Metabolism of Terpenoids and Polyketides (*P* < 0.05).

## Discussion

Wagyu cattle are highly valued for their breed-specific capacity to produce marbled beef. However, this trait is associated with complex rearing procedures and high production costs [11]. In contrast, Holstein cattle, though primarily selected for milk production, have demonstrated potential for marbled beef production in male calves [12]. Previous studies have demonstrated that rumen microbiota play a pivotal role in the nutrition and energy metabolism of ruminants, participating in the degradation of dietary carbohydrates such as cellulose and starch [13, 14], and converting dietary nitrogen sources into microbial protein [15, 16], thereby supplying nutrients essential for host growth and development. In this study, we characterized the rumen microbial composition and functional gene profiles in Wagyu and Holstein calves from 3 to 6 months of age, a critical window for microbial colonization and functional maturation [6–8].

KEGG-based functional profiling revealed that, at 3 months of age, rumen microbial gene distributions were largely similar across breeds. In Wagyu calves, at the same age, inter-breed differences in rumen microbial functions were mainly characterized by a higher relative abundance of genes related to genetic information processing and cellular processes, such as translation, folding, sorting and degradation, replication and repair, and transcription. In contrast, Holstein calves exhibited a relative enrichment of genes involved in metabolic pathways, including carbohydrate metabolism, amino acid metabolism, energy metabolism, and lipid metabolism. These findings are consistent with those reported by Dong et al. [17] and Malik et al. [18], who compared rumen microbiota and functional profiles between Bohai Black cattle and Holstein dairy cattle, and between crossbred bulls and water buffaloes, respectively.

Firmicutes and Bacteroidetes remained the dominant phyla, and *Prevotella* was the predominant genus in both breeds. These findings are consistent with those reported by Du et al. [19], Huuki et al. [20], and Ahmad et al. [21] in studies examining age-related changes in calf rumen microbiota. The phylum Firmicutes includes genera such as Lactobacillus, Butyrivibrio, and Streptococcus, while Bacteroidetes includes Prevotella, Bacteroides, and Parabacteroides. These groups synergistically degrade fiber and complex polysaccharides in the rumen to support host energy supply [22]. It has also been reported that an increased Firmicutes-to-Bacteroidetes ratio is associated with obesity and intestinal inflammation [23–25]. In this study, the Firmicutes-to-Bacteroidetes ratio decreased with age in both breeds and was consistently higher in Holstein calves than in Wagyu calves at the same age, although the factors influencing this difference warrant further investigation.

### Breed-Specific succession patterns in rumen microbial community structure in calves

At the phylum level, both Wagyu (Wagyu) and Holstein calves were predominantly colonized by Firmicutes and Bacteroidetes. However, Wagyu calves exhibited a significant age-related increase in the relative abundance of Fibrobacteres and Euryarchaeota, reflecting an adaptive response to enhanced fiber degradation and methanogenesis demands during development. In contrast, Holstein calves maintained a relatively stable phylum-level and genus-level composition, likely attributable to their genetic background shaped by high-concentrate feeding regimens typical of dairy production systems [26]. Wagyu calves showed a gradual decrease in *Prevotella* with concurrent increases in *Fibrobacter* and *Methanobrevibacter*, indicating a functional transition from lactate metabolism during weaning to fiber degradation in later stages. Meanwhile, Holstein calves displayed a stable genus composition with consistently high levels of *Butyrivibrio* and *Methanogens*, supporting efficient short-chain fatty acid production [27]. These breed-specific differences likely arise from genetically driven host-microbiota interactions, where Wagyu calves may optimize fiber utilization via microbial amplification and selective colonization, whereas Holstein calves preserve energy metabolism efficiency through a more stable microbial community.

### Breed-Divergent mechanisms underlying rumen microbial diversity and functional maturation

Alpha diversity indices (Shannon, ACE, and Chao1) increased significantly with age in Wagyu calves, indicating enhanced microbial community complexity and colonization by rare taxa such as fiber-degrading bacteria, which promotes functional differentiation. In contrast, Holstein calves exhibited relatively stable alpha diversity throughout the study, suggesting earlier establishment of a steady-state microbiome. Beta diversity analyses further revealed that microbial community similarity varied substantially with age in Wagyu calves, reflecting dynamic selective processes. Conversely, Holstein calves showed high microbial community similarity over time. These patterns are influenced by both genetic background, such as Wagyu cattle mature slowly and need extended fattening for premium marbling, whereas young Holstein cattle prepare for lactation early [28–30]. Thus, the differences found between the two breeds could be related to metabolic differences.

### Functional gene succession reveals Breed-Specific metabolic strategies

Functional gene annotation predicted distinct metabolic trajectories between the breeds. In Wagyu calves, the relative abundance as determined by metagenomic of genes associated with metabolism decreased at 6 months, whereas the relative abundance of genes involved in genetic information processing and cellular processes increased. This shift likely reflects a microbial transition from substrate degradation toward proliferation and adaptive evolution, exemplified by genomic expansion in taxa such as *Fibrobacter*. Holstein calves, in contrast, the clearest trend was a significant enrichment of genes in pathways related to metabolism, with persistent activity in carbohydrate and amino acid metabolism pathways likely supporting early growth and future milk fat synthesis. Key pathway analysis indicated that Wagyu calves were enriched in pathways related to translation and cell motility, facilitating microbial reconstruction of enzymatic systems for fiber substrate utilization [31–33]. Holstein calves showed enrichment in lipid metabolism and xenobiotic degradation pathways, possibly suggesting enhanced adaptability to complex dietary components.

### LEfSe biomarkers and potential associations with host productive performance

LEfSe analysis screened breed-specific microbial biomarkers with implications for production efficiency and environmental impact. Wagyu calves exhibited sustained enrichment of Fibrobacter and Methanobrevibacter, supporting efficient fiber degradation and methane production that possibly enhance weight gain but contribute to greenhouse gas emissions [34]. In contrast, Holstein calves maintained stable populations of Butyrivibrio and methanogens, which help maintain ruminal pH via butyrate production, a key intestinal energy source, thereby promoting lactation performance [35]. These insights suggest potential targeted interventions, such as substrate-competing fiber-degrading probiotic supplementation in Wagyu calves, and possibly targeted methanogen inhibition, to optimize production while mitigating environmental impacts [36, 37].

### Study limitations and future directions

We are aware that this research has some limitations. First, the small sample size (*n* = 5 per group) may limit statistical power and increase susceptibility to individual variation. While effect sizes were calculated to contextualize our findings, larger studies are needed to improve the generalizability of these conclusions. Second, the metagenomic sequencing provides only functional predictions rather than a true external validation. Additionally, slaughtering calves for sampling is inhumane and wasteful, so body-weight growth serves as readily accessible external validation is understandable. However, despite breed accounting for much of the difference in IBW and FBW between Wagyu and Holstein calves, average daily gain did not differ significantly. Thus, the weight data alone are insufficient for validation. Future research should increase sample size and extend the observation period to fattening stages: First, Wagyu calves could receive a wider array of grain-based concentrates to accelerate rumen microbial maturation and shift metabolic pathways toward earlier physiological readiness. Second, building on our straight-fattening protocol for Holstein steers that produces marbled beef, we will test whether inoculating these steers with Wagyu calf rumen fluid preserves microbial diversity and the associated functional pathways, and quantify the impact on beef quality.

## Conclusion

In summary, this study speculates novel insights into breed-specific differences in the rumen microbiota of Wagyu and Holstein calves during early development. Wagyu calves may undergo dynamic microbial restructuring, with a progressive shift from metabolic to adaptive genetic functions, while Holstein calves maintained stable communities with efficient metabolic function. These structural and functional characteristics of rumen microbiota may provide theoretical references for early nutritional interventions.

## Supplementary Information

Supplementary Material 1.

## Data Availability

The datasets generated and/or analysed during the current study are available in the National Genomics Data Center (NGDC) repository, under the BioProject accession PRJCA041230. The data can be accessed via the following link:  https://ngdc.cncb.ac.cn/bioproject/browse/PRJCA041230.

## Abbreviations

KEGG: Kyoto Encyclopedia of Genes and Genomes
LEfSe: Linear Discriminant Analysis Effect Size
PCoA: Principal Coordinates Analysis
ACE: Abundance–based Coverage Estimator
ORFs: Open Reading Frames
VFAs: Volatile Fatty Acids
IACUC: Institutional Animal Care and Use Committee
DNA: Deoxyribonucleic Acid
PCR: Polymerase Chain Reaction
FDR: False Discovery Rate
PERMANOVA: Permutational Multivariate Analysis of Variance
ANOVA: Analysis of Variance
NR: Non–Redundant (database)
