# Supplementary material for: Comparative study on the rumen microbial communities and functions between Wagyu and Holstein calves
Source: BMC Genomics. 2025 Dec 4;27:20. doi: 10.1186/s12864-025-12392-1 (PMC12781348; doi:10.1186/s12864-025-12392-1)
Supplement: Supplementary file 1 — Supplementary Material 1. [file 12864_2025_12392_MOESM1_ESM.docx]

Table S1. Growth performance of Wagyu and Holstein calves during 3-6 months of age

| Items | Wagyu | Holstein | *P*-value |
| --- | --- | --- | --- |
| 3 months of age weight (kg) | 83.1±2.19 | 98.32±2.45 | < 0.001^**^ |
| 4 months of age weight (kg) | 118.4±3.41 | 121.48±4.36 | 0.249 |
| 5 months of age weight (kg) | 148.14±5.91 | 157.98±4.67 | 0.019^*^ |
| 6 months of age weight (kg) | 187.8±6.26 | 196.4±4.34 | 0.036^*^ |
| Average daily gain (kg) | 1.08±0.09 | 1.07±0.06 | 0.958 |

Table S2. Phylum-level distribution of rumen microbiota (Based on metagenomic annotations with a relative abundance threshold of > 0.1%)

| **Items (%)** | **From 3 to 6 months of age** | | | |
| --- | --- | --- | --- | --- |
|  | **W3** | **W4** | **W5** | **W6** |
| Bacteroidetes | 41.75 | 46.45 | 39.48 | 40.89 |
| Firmicutes | 40.39 | 36.70 | 39.81 | 35.18 |
| Proteobacteria | 3.96 | 4.21 | 5.63 | 4.93 |
| Fibrobacteres | 2.53 | 1.41 | 3.50 | 4.63 |
| Euryarchaeota | 1.25 | 2.20 | 2.51 | 3.15 |
| Actinobacteria | 3.36 | 1.95 | 1.50 | 1.29 |
| Spirochaetes | 2.58 | 0.79 | 1.28 | 1.64 |
| Chytridiomycota | 0.48 | 0.34 | 0.65 | 1.25 |
| Lentisphaerae | 0.10 | 0.77 | 0.81 | 0.91 |
| Uroviricota | 0.53 | 0.74 | 0.74 | 0.43 |
| Candidatus_Melainabacteria | 0.38 | 0.98 | 0.22 | 0.36 |
| Ascomycota | 0.37 | 0.19 | 0.41 | 0.90 |
| Mucoromycota | 0.33 | 0.22 | 0.41 | 0.83 |
| Chlamydiae | 0.30 | 0.53 | 0.44 | 0.22 |
| Verrucomicrobia | 0.06 | 0.32 | 0.36 | 0.40 |
| Tenericutes | 0.23 | 0.24 | 0.28 | 0.30 |
| Basidiomycota | 0.14 | 0.08 | 0.15 | 0.32 |
| Zoopagomycota | 0.12 | 0.08 | 0.15 | 0.31 |
| Planctomycetes | 0.04 | 0.22 | 0.19 | 0.18 |
| Fusobacteria | 0.22 | 0.17 | 0.10 | 0.11 |
| Elusimicrobia | 0.13 | 0.14 | 0.11 | 0.20 |
| Chloroflexi | 0.07 | 0.17 | 0.12 | 0.12 |
| Candidatus_Saccharibacteria | 0.05 | 0.17 | 0.08 | 0.12 |
| Others | 0.64 | 0.92 | 1.05 | 1.33 |
| Total | 100.00 | 100.00 | 100.00 | 100.00 |
|  | **H3** | **H4** | **H5** | **H6** |
| Firmicutes | 46.59 | 47.13 | 44.48 | 42.79 |
| Bacteroidetes | 41.44 | 40.58 | 43.35 | 45.45 |
| Euryarchaeota | 3.92 | 4.00 | 3.64 | 3.27 |
| Actinobacteria | 2.03 | 2.22 | 1.79 | 1.73 |
| Proteobacteria | 1.79 | 1.76 | 1.80 | 1.65 |
| Lentisphaerae | 0.89 | 1.02 | 0.97 | 0.91 |
| Spirochaetes | 0.56 | 0.54 | 0.81 | 0.92 |
| Verrucomicrobia | 0.38 | 0.44 | 0.42 | 0.38 |
| Planctomycetes | 0.38 | 0.38 | 0.39 | 0.34 |
| Uroviricota | 0.33 | 0.28 | 0.41 | 0.47 |
| Fibrobacteres | 0.30 | 0.27 | 0.33 | 0.47 |
| Tenericutes | 0.23 | 0.24 | 0.33 | 0.33 |
| Chlamydiae | 0.24 | 0.22 | 0.29 | 0.33 |
| Chloroflexi | 0.13 | 0.14 | 0.12 | 0.12 |
| Fusobacteria | 0.10 | 0.10 | 0.11 | 0.10 |
| Others | 0.68 | 0.68 | 0.77 | 0.75 |
| Total | 100.00 | 100.00 | 100.00 | 100.00 |

Table S3. Genus-level distribution of rumen microbiota (Based on metagenomic annotations with a relative abundance threshold of > 0.1%)

| **Items (%)** | **From 3 to 6 months of age** | | | |
| --- | --- | --- | --- | --- |
|  | **W3** | **W4** | **W5** | **W6** |
| *Prevotella* | 38.63 | 40.26 | 34.72 | 34.69 |
| *Bacteroides* | 4.00 | 5.60 | 5.33 | 5.34 |
| *Fibrobacter* | 3.39 | 2.11 | 5.41 | 7.07 |
| *Ruminococcus* | 3.38 | 3.64 | 3.81 | 3.52 |
| *Clostridium* | 2.82 | 3.02 | 3.48 | 3.12 |
| *Methanobrevibacter* | 1.54 | 2.89 | 3.53 | 4.29 |
| *Butyrivibrio* | 1.53 | 3.30 | 3.16 | 2.96 |
| *Treponema* | 3.38 | 0.74 | 1.64 | 2.13 |
| *Eubacterium* | 1.78 | 1.59 | 1.68 | 1.34 |
| *Intestinibaculum* | 5.07 | 0.01 | 0.02 | 0.02 |
| *Selenomonas* | 3.45 | 1.01 | 0.33 | 0.27 |
| *Sarcina* | 0.98 | 0.94 | 2.07 | 1.33 |
| *Succinivibrio* | 1.00 | 0.91 | 1.81 | 1.37 |
| *Alistipes* | 0.80 | 1.00 | 1.03 | 0.92 |
| *Succiniclasticum* | 0.65 | 0.87 | 0.95 | 1.18 |
| *Staphylococcus* | 0.23 | 2.68 | 0.36 | 0.13 |
| *Paludibacter* | 0.35 | 1.10 | 0.65 | 0.81 |
| *Pseudobutyrivibrio* | 0.18 | 0.92 | 0.84 | 0.88 |
| *Parabacteroides* | 0.47 | 0.69 | 0.62 | 0.62 |
| *Faecalibacterium* | 0.43 | 0.57 | 0.82 | 0.62 |
| *Blautia* | 0.57 | 0.59 | 0.71 | 0.53 |
| *Ruminobacter* | 0.27 | 0.18 | 1.16 | 0.73 |
| *Phocaeicola* | 0.46 | 0.63 | 0.55 | 0.57 |
| *Chlamydia* | 0.41 | 0.71 | 0.65 | 0.29 |
| *Oscillibacter* | 0.30 | 0.42 | 0.45 | 0.84 |
| *Lachnoclostridium* | 0.52 | 0.48 | 0.55 | 0.46 |
| *Oribacterium* | 0.35 | 0.49 | 0.54 | 0.53 |
| *Roseburia* | 0.67 | 0.39 | 0.40 | 0.33 |
| *Coprobacillus* | 0.22 | 0.28 | 0.58 | 0.63 |
| *Succinimonas* | 0.25 | 0.12 | 0.50 | 0.62 |
| *Olsenella* | 0.31 | 0.43 | 0.39 | 0.32 |
| *Succinatimonas* | 0.52 | 0.19 | 0.43 | 0.25 |
| *Pseudoscardovia* | 1.22 | 0.00 | 0.00 | 0.00 |
| *Azospirillum* | 0.35 | 0.57 | 0.06 | 0.14 |
| *Anaerolactibacter* | 0.92 | 0.06 | 0.07 | 0.06 |
| *Pseudomonas* | 0.25 | 0.44 | 0.20 | 0.24 |
| *Flavonifractor* | 0.19 | 0.29 | 0.34 | 0.26 |
| *Desulfovibrio* | 0.26 | 0.18 | 0.34 | 0.29 |
| *Paraprevotella* | 0.23 | 0.27 | 0.23 | 0.29 |
| *Megasphaera* | 0.72 | 0.06 | 0.08 | 0.06 |
| *Parafannyhessea* | 0.74 | 0.05 | 0.05 | 0.04 |
| *Pseudoprevotella* | 0.19 | 0.26 | 0.20 | 0.23 |
| *Neocallimastix* | 0.15 | 0.10 | 0.22 | 0.41 |
| *Paenibacillus* | 0.15 | 0.22 | 0.28 | 0.23 |
| *Enterocloster* | 0.18 | 0.22 | 0.24 | 0.18 |
| *Enterococcus* | 0.11 | 0.41 | 0.16 | 0.12 |
| *Fusobacterium* | 0.28 | 0.24 | 0.13 | 0.14 |
| *Subdoligranulum* | 0.15 | 0.22 | 0.25 | 0.18 |
| *Bacillus* | 0.14 | 0.27 | 0.21 | 0.16 |
| *Dialister* | 0.68 | 0.01 | 0.03 | 0.02 |
| *Acetobacter* | 0.25 | 0.27 | 0.04 | 0.19 |
| *Schwartzia* | 0.14 | 0.28 | 0.22 | 0.14 |
| *Streptococcus* | 0.17 | 0.28 | 0.17 | 0.13 |
| *Anaerotruncus* | 0.15 | 0.19 | 0.23 | 0.18 |
| *Flavobacterium* | 0.13 | 0.25 | 0.18 | 0.17 |
| *Butyricicoccus* | 0.15 | 0.19 | 0.21 | 0.16 |
| *Piromyces* | 0.12 | 0.09 | 0.17 | 0.33 |
| *Sharpea* | 0.58 | 0.02 | 0.03 | 0.02 |
| *Coprococcus* | 0.18 | 0.16 | 0.18 | 0.14 |
| *Slackia* | 0.11 | 0.21 | 0.18 | 0.14 |
| *Methanosphaera* | 0.05 | 0.14 | 0.16 | 0.28 |
| *Dorea* | 0.16 | 0.14 | 0.17 | 0.14 |
| *Tannerella* | 0.10 | 0.20 | 0.15 | 0.16 |
| *Dysgonomonas* | 0.08 | 0.19 | 0.15 | 0.14 |
| *Mediterraneibacter* | 0.17 | 0.13 | 0.14 | 0.10 |
| *Bifidobacterium* | 0.20 | 0.09 | 0.12 | 0.13 |
| *Barnesiella* | 0.12 | 0.16 | 0.13 | 0.13 |
| *Mycoplasma* | 0.10 | 0.14 | 0.11 | 0.15 |
| *Rozella* | 0.08 | 0.06 | 0.12 | 0.23 |
| *Intestinimonas* | 0.08 | 0.13 | 0.14 | 0.14 |
| *Candidatus_Nanosyncoccus* | 0.05 | 0.20 | 0.10 | 0.14 |
| *Mitsuokella* | 0.37 | 0.04 | 0.03 | 0.02 |
| *Klebsiella* | 0.04 | 0.38 | 0.03 | 0.02 |
| *Porphyromonas* | 0.09 | 0.16 | 0.12 | 0.11 |
| *Endomicrobium* | 0.12 | 0.08 | 0.07 | 0.21 |
| *Odoribacter* | 0.07 | 0.18 | 0.12 | 0.10 |
| *Brachyspira* | 0.07 | 0.24 | 0.10 | 0.06 |
| *Anaeroplasma* | 0.12 | 0.07 | 0.14 | 0.15 |
| *Pseudoflavonifractor* | 0.09 | 0.11 | 0.14 | 0.12 |
| *Anaeromassilibacillus* | 0.10 | 0.11 | 0.15 | 0.10 |
| *Collinsella* | 0.13 | 0.13 | 0.12 | 0.08 |
| *Acinetobacter* | 0.05 | 0.26 | 0.05 | 0.06 |
| *Mogibacterium* | 0.08 | 0.09 | 0.15 | 0.13 |
| *Others* | 9.71 | 11.96 | 13.41 | 13.75 |
| Total | 100.00 | 100.00 | 100.00 | 100.00 |
|  | **H3** | **H4** | **H5** | **H6** |
| *Prevotella* | 38.63 | 38.56 | 38.89 | 39.54 |
| *Bacteroides* | 6.21 | 5.94 | 6.84 | 7.20 |
| *Butyrivibrio* | 5.84 | 6.11 | 5.00 | 4.77 |
| *Methanobrevibacter* | 5.67 | 5.81 | 5.25 | 4.80 |
| *Sarcina* | 3.81 | 3.70 | 3.62 | 3.41 |
| *Clostridium* | 3.42 | 3.40 | 3.57 | 3.37 |
| *Ruminococcus* | 3.03 | 3.12 | 3.18 | 2.96 |
| *Eubacterium* | 1.85 | 1.91 | 1.80 | 1.73 |
| *Succiniclasticum* | 1.61 | 1.67 | 1.44 | 1.38 |
| *Alistipes* | 1.36 | 1.27 | 1.45 | 1.65 |
| *Pseudobutyrivibrio* | 1.11 | 1.00 | 1.03 | 0.87 |
| *Treponema* | 0.75 | 0.72 | 1.13 | 1.32 |
| *Faecalibacterium* | 1.00 | 1.00 | 0.94 | 0.94 |
| *Blautia* | 0.86 | 0.86 | 0.81 | 0.75 |
| *Phocaeicola* | 0.78 | 0.73 | 0.83 | 0.87 |
| *Lachnoclostridium* | 0.77 | 0.78 | 0.73 | 0.70 |
| *Oribacterium* | 0.75 | 0.74 | 0.65 | 0.61 |
| *Parabacteroides* | 0.56 | 0.55 | 0.63 | 0.70 |
| *Fibrobacter* | 0.46 | 0.41 | 0.50 | 0.71 |
| *Desulfobulbus* | 0.52 | 0.56 | 0.53 | 0.45 |
| *Oscillibacter* | 0.52 | 0.53 | 0.50 | 0.49 |
| *Paraprevotella* | 0.42 | 0.43 | 0.55 | 0.52 |
| *Roseburia* | 0.44 | 0.44 | 0.42 | 0.40 |
| *Chlamydia* | 0.37 | 0.33 | 0.44 | 0.51 |
| *Barnesiella* | 0.34 | 0.35 | 0.37 | 0.42 |
| *Desulfovibrio* | 0.38 | 0.40 | 0.35 | 0.31 |
| *Schwartzia* | 0.36 | 0.40 | 0.34 | 0.32 |
| *Methanosphaera* | 0.39 | 0.39 | 0.35 | 0.26 |
| *Olsenella* | 0.35 | 0.39 | 0.32 | 0.31 |
| *Pseudoprevotella* | 0.31 | 0.31 | 0.34 | 0.36 |
| *Slackia* | 0.32 | 0.36 | 0.29 | 0.28 |
| *Selenomonas* | 0.32 | 0.34 | 0.30 | 0.28 |
| *Flavonifractor* | 0.30 | 0.30 | 0.29 | 0.29 |
| *Mycoplasma* | 0.26 | 0.27 | 0.34 | 0.26 |
| *Enterocloster* | 0.25 | 0.25 | 0.25 | 0.26 |
| *Anaerotruncus* | 0.25 | 0.25 | 0.25 | 0.24 |
| *Mogibacterium* | 0.23 | 0.26 | 0.23 | 0.22 |
| *Subdoligranulum* | 0.23 | 0.23 | 0.23 | 0.24 |
| *Paenibacillus* | 0.23 | 0.23 | 0.23 | 0.23 |
| *Paludibacter* | 0.18 | 0.18 | 0.23 | 0.30 |
| *Coprococcus* | 0.23 | 0.22 | 0.21 | 0.20 |
| *Tannerella* | 0.20 | 0.19 | 0.22 | 0.25 |
| *Sporobacter* | 0.22 | 0.23 | 0.20 | 0.19 |
| *Coprobacter* | 0.19 | 0.19 | 0.21 | 0.23 |
| *Pseudoflavonifractor* | 0.21 | 0.21 | 0.20 | 0.19 |
| *Dorea* | 0.21 | 0.21 | 0.19 | 0.18 |
| *Anaeromassilibacillus* | 0.17 | 0.18 | 0.18 | 0.17 |
| *Dietzia* | 0.20 | 0.18 | 0.23 | 0.08 |
| *Mobilibacterium* | 0.17 | 0.18 | 0.17 | 0.16 |
| *Pseudomonas* | 0.23 | 0.10 | 0.21 | 0.12 |
| *Butyricicoccus* | 0.16 | 0.16 | 0.16 | 0.16 |
| *Anaerolactibacter* | 0.18 | 0.18 | 0.15 | 0.12 |
| *Denitrobacterium* | 0.17 | 0.20 | 0.13 | 0.13 |
| *Streptococcus* | 0.16 | 0.16 | 0.15 | 0.16 |
| *Intestinimonas* | 0.15 | 0.16 | 0.14 | 0.14 |
| *Fusobacterium* | 0.14 | 0.14 | 0.16 | 0.14 |
| *Bacillus* | 0.14 | 0.13 | 0.14 | 0.15 |
| *Mediterraneibacter* | 0.14 | 0.14 | 0.14 | 0.13 |
| *Gordonibacter* | 0.14 | 0.17 | 0.11 | 0.12 |
| *Alloprevotella* | 0.12 | 0.12 | 0.14 | 0.15 |
| *Hungatella* | 0.13 | 0.13 | 0.13 | 0.13 |
| *Eggerthella* | 0.13 | 0.15 | 0.11 | 0.11 |
| *Flavobacterium* | 0.11 | 0.11 | 0.13 | 0.15 |
| *Victivallis* | 0.12 | 0.13 | 0.13 | 0.12 |
| *Odoribacter* | 0.11 | 0.11 | 0.13 | 0.15 |
| *Dysgonomonas* | 0.11 | 0.11 | 0.13 | 0.14 |
| *Porphyromonas* | 0.11 | 0.11 | 0.12 | 0.14 |
| *Hornefia* | 0.12 | 0.12 | 0.11 | 0.10 |
| *Acutalibacter* | 0.11 | 0.11 | 0.11 | 0.11 |
| *Lachnospira* | 0.11 | 0.11 | 0.11 | 0.11 |
| *Anaerovibrio* | 0.11 | 0.12 | 0.10 | 0.09 |
| *Bifidobacterium* | 0.11 | 0.11 | 0.10 | 0.10 |
| *Marvinbryantia* | 0.10 | 0.11 | 0.10 | 0.09 |
| *Others* | 9.51 | 9.57 | 9.60 | 10.04 |
| Total | 100.00 | 100.00 | 100.00 | 100.00 |
